# Genetic Testing by Sports Medicine Physicians in the United States: Attitudes, Experiences, and Knowledge

**DOI:** 10.3390/sports6040145

**Published:** 2018-11-12

**Authors:** Eleanor Taranto, Michael Fishman, Holly Benjamin, Lainie Ross

**Affiliations:** 1 Pritzker School of Medicine, University of Chicago, Chicago, IL 60637, USA; etaranto@uchicago.edu (E.T.); mfishman@uchicago.edu (M.F.); 2 Department of Pediatrics, University of Chicago Medicine, Chicago, IL 60637, USA; hbenjami@bsd.uchicago.edu; 3 Department of Orthopedic Surgery and Rehabilitation Medicine, University of Chicago Medicine, Chicago, IL 60637, USA; 4 MacLean Center for Clinical Medical Ethics, University of Chicago, Chicago, IL 60637, USA

**Keywords:** genetic testing, sports medicine, direct-to-consumer testing, athletic performance, sickle cell trait

## Abstract

It remains unknown whether and how sports medicine physicians currently utilize genetic testing in their clinical practice. This study sought to assess knowledge of, experience with, and attitudes towards genetic testing by sports medicine physicians in the United States (US). An email with a survey hyperlink was distributed twice to members of the American Medical Society for Sports Medicine (AMSSM) listserv in September 2016, with approximately a 10% response rate. Questions focused on knowledge of, experience with, and attitudes towards testing for different genes related to sports proficiency, injury risk, and disease risk. Few AMSSM physicians believe that genetic testing to adapt training (12%) or to choose a sport (2%) is ready for clinical adoption. Most respondents self-reported minimal knowledge about, and limited experience with, genetic testing. The main exception was screening for sickle cell trait (SCT) for which most (84%) reported moderate/significant/expert knowledge and over two-thirds had ordered testing. Although most respondents thought it appropriate to counsel and test for health conditions associated with cardiac and connective tissue disorders in the setting of a positive family history, only a minority had been asked to do so. Five or fewer respondents (2%) had been asked to test for performance-associated variants (Angiotensin Converting Enzyme *(ACE) II* and Alpha-Actinin 3 (*ACTN3*)), and five or fewer (2%) would recommend changes based on the results. Our study provides a baseline of current US sports medicine physicians’ minimal experiences with, and knowledge of, genetic testing. The findings of our study indicate that sports medicine physicians require further genetics education as it relates to sports and exercise in order to be prepared to competently engage with their patients and to develop sound professional organizational policies.

## 1. Introduction

There are many reasons why health care providers in the United States (US) involved in sports medicine and exercise physiology may be interested in the potential role of genetics and genetic testing—whether to help predict athletic performance, to modify athletic training, or to prevent injury [1,2,3]. While the clinical utility of genetic testing in this sphere is still in its infancy, research is ongoing [1,2,3]. 

One of the first correlations between genetics and exercise was identified half a century ago when Jones and colleagues showed an association between sickle cell trait (SCT), exertional heat illness (EHI), and sudden death in the basic training of new US military recruits [4]. Studies over the next 40 years confirmed the association of SCT with EHI or exertional rhabdomyolysis [5,6,7,8,9]. In response to the death of a Rice University football player with SCT in 2006 [10], the National Collegiate Athletic Association (NCAA) implemented a policy to screen all incoming athletes for SCT. This policy is not without controversy, with some organizations (e.g., National Athletic Trainers’ Association (NATA), College of American Pathologists (CAP)) supporting SCT screening of athletes to reduce EHI and sudden death [11,12], and other organizations, (including the American Society for Hematology (ASH) [13], The Secretary’s Advisory Committee on Heritable Disorders in Newborns and Children (SACHDNC) [14], and the American Academy of Pediatrics (AAP) [14]) opposing it—as it remains unclear how, and if, medical recommendations regarding participation and training should be modified based on SCT status [13]. 

Another genetic variant relevant for sports medicine is Apolipoprotein E4 (*Apoe4*). Recently, researchers have identified *Apoe4* as a potential risk factor that negatively influences recovery outcomes following concussion. Sports-related concussions are a major source of traumatic brain injury (TBI) in the pediatric population, and the long-term sequelae of repeated concussions are largely unknown [15]. Recently, researchers have begun to focus on *Apoe4* as a risk factor for worse recovery from concussion, but its precise role in the recovery mechanism—and how that role may vary across gender, ethnicity, and age—remains unknown [16]. Although ongoing research is examining the association [17,18], there is currently no evidence to support screening athletes for *Apoe4*—nor is there support for modifying participation or training based on the results. 

Other genes are also potentially relevant to sports medicine. Researchers have documented associations between various genes and health conditions that may impact sports participation; hypertrophic cardiomyopathy (HCM), connective tissue disorders, long QT syndrome (LQTS), cardiac arrhythmias, and collagen, type I, alpha 1 (*COL1A1*) variants associated with tendon rupture can all have a genetic basis and can cause symptoms or injury during exertion. Genetic testing may be considered because an athlete has a positive family history or symptoms. Several other genes have been hypothesized to contribute to athletic performance by providing a phenotypic advantage. Two such genes are Alpha-Actinin 3 (*ACTN3*), which has variants expressed in fast twitch fibers and is highly expressed in sprinters, and Angiotensin Converting Enzyme *(ACE) II*, whose variants may be associated with improved performance in endurance sports [19]. However, their utility in predicting athletic ability remains highly questionable [20,21]. To date, most of these studies have utilized small sample sizes and focused on elite athletes [22]. For most of these genes, replication research has only inconsistently confirmed the causal link between particular genes and different types of athletic performance, let alone the predictive power of gene variants in the sports-choosing sphere [23,24,25]. 

Although current evidence does not support the use of genetic testing for the prediction or optimization of athletic ability [20,26], direct-to-consumer (DTC) companies in the United States (US) are marketing their services to parents who are encouraged to use the information to make decisions about placing children into particular sports—sometimes at an extremely young age—as well as the level of commitment to devote to such a sport [26]. The extent to which US consumers discuss whether to undergo DTC genetic testing with sports medicine physicians, and whether they discuss their results, is unknown. Other unknowns include physicians familiarity with, and support for, such testing, and their ability to interpret the results and counsel and educate families on their meanings. There is currently a lack of baseline data about the attitudes, experiences, and knowledge of sports medicine physicians regarding genetic testing, in spite of continued interest from the media, public, and commercial ventures alike in linking personalized medicine to sports performance. We therefore sought to evaluate the self-described knowledge, attitudes, and experiences of physicians in the American Medical Society for Sports Medicine (AMSSM) towards genetic testing.

## 2. Materials and Methods

### 2.1. Participants

An email with a survey hyperlink was distributed twice in September 2016 to approximately 2157 members on the AMSSM listserv. The exact number of email recipients is unknown, since not all members have emails, some opt out of the listserv, and others receive listserv emails to more than one address. Data were collected anonymously using the electronic software Research Electronic Data Capture (REDCAP).

### 2.2. Survey

The survey included Likert-scale questions and multiple-choice questions related to genetic testing and its use in sports, including both (1) the frequency of genetic testing requests for particular traits and variants thought to be related to sports proficiency and injury risk; and (2) the attitudes and self-rated knowledge of physicians towards performing such testing on pediatric patients and making recommendations based on the results. Short descriptions of each condition/variant and their associations with disease/injury risk or athletic performance were provided on the survey (the survey is available from the corresponding author upon request). Additional questions focused on SCT, *Apoe4*, and DTC testing policies and practices, and demographics. The survey was determined to be exempt by the University of Chicago Institutional Review Board, with consent implied by participation.

### 2.3. Data Analysis

Statistical analysis was performed using SPSS (version 24.0; IBM Corp, Armonk, NY, USA). Standard descriptive summaries (frequencies for categorical variables) were obtained, and comparative statistics of categorical variables (Chi-squared tests) were performed using a significance level *p* < 0.05. 

### 2.4. Collapsed Variables

For data analysis, some variables were collapsed. Questions using a 4-option Likert-scale were collapsed into two: “Definitely Yes” and “Probably Yes” were collapsed into “Yes”, and “Definitely No” and “Probably No” were collapsed into “No”. When asked whether they had been asked to perform genetic testing, the answer choices “Yes” and “Yes. Referred to genetic counselors.” were collapsed into “Yes”. For the question regarding whether physicians thought it was appropriate to counsel/test children interested in sports for particular genes, the two positive variables “Yes. Any child” and “Yes. But only with family history” were collapsed into “Yes”, distinct from “No” and “Don’t know”. For the question whether physicians would recommend changes in sports participation based on genetic findings, the responses “No” and “Don’t know” were combined as distinct from “Yes”. The seven response options for “Years in Practice” (“Still in Training”, “0 to <5 years”, “5 to <10 years”, “10 to <15 years”, “15 to <20 years”, “20 to <25 years”, and “25 or more years”) were collapsed into “Still in training”, “Zero to less than 10 years in practice”, and “Ten years or more in practice”. 

## 3. Results

Two hundred and sixteen complete or partial surveys (majority complete) were collected (~10% response rate). Denominators for each question are provided due to missing data. Demographics appear in Table 1.

The majority of respondents were male (74%), white (86%), trained in family practice medicine (70%), and completed a sports medicine fellowship (90%). Most see a mix of children and adults, although 64 (31%) see mostly children <18 years. Very few (n = 14) have sought out DTC genetic testing for themselves, and none have sought out DTC testing for their children. 

As shown in Table 2, respondents self-described as having a low level of knowledge about genetic testing overall (57% reporting minimal knowledge and 41% moderate knowledge). 

Virtually all physicians (207, 98%) believe that multiplex genetic testing to influence which sport a potential athlete pursues is **not** ready for prime time. The vast majority (185, 88%) also do not believe that the use of multiplex genetic testing to influence an athlete’s training program is ready for prime time. In fact, most respondents (172, 82%) reported that no patients had ever requested athletic-performance-related genetic testing, and most have never had a patient request that they review DTC genetic test results (165, 79%). Among those physicians receiving testing requests, more are receiving requests from parents (56%) rather than from athletes (19%). 

Despite low general knowledge, respondents did express familiarity with SCT (84% have more than minimal knowledge), and over two-thirds had been asked to test a patient—whether because of the NCAA mandate, clinical judgement, or because of patient request (See Table 3). 

The majority of respondents were in favor of the NCAA SCT policy, although only half would recommend changes in sports participation based on these results. Serving as a college team physician did not significantly influence whether or not respondents agreed with the NCAA policy: 68% (127/186) of team physicians versus 75% (15/20) of non-team physicians agreed with the NCAA policy (*p* = 0.537). However, more physicians who agreed with the NCAA policy **would** recommend changes in sports participation based on SCT (84/133, 63%) compared with physicians who did not agree (22/63, 35%) (*p* = 0.001). 

In contrast with knowledge about SCT, only 28% of respondents self-described their knowledge of *Apoe4* as moderate or significant (see Table 4). 

However, requests for *Apoe4* testing were uncommon, and 52% of physicians did not think it appropriate to test children for *Apoe4*—with another 24% who were not sure. While just six individuals (3%) would recommend *Apoe4* testing in order to make decisions about continued sports participation after a patient suffers a concussion, another 56 (27%) would perform *Apoe4* testing if a parent or patient requested it. Nevertheless, only 36 (18%) would change return-to-play recommendations based on the results. Virtually all who **would not** test for *Apoe4* after a concussion said that the results **would not** change their recommendations (131/137, 96%), while only 32/62 (52%) of those who **would** test for *Apoe4* after a concussion said that the results **would not** change their recommendations (*p* < 0.001). 

Respondents were asked about seven other conditions or genetic variants, five of which were associated with health risks (connective tissue disorders, HCM, LQTS, cardiac arrhythmias, and *COL1A1* variants) and two of which were associated with performance (*ACE II* and *ACTN 3* variants) (see Table 5). 

Less than half of respondents had been asked to do genetic testing for any of these variants, although it was more common to have been asked about four of the five conditions that pose health risks, and for those four conditions, a majority thought it would be appropriate to counsel and test children. In contrast, less than 15% of respondents thought it would be appropriate to counsel or test children for the genetic variants associated with performance, with five or fewer respondents (2%) stating that they would recommend changes based on the results. 

## 4. Discussion

Despite lay and professional talk about precision and personalized medicine [27,28,29], genetic testing has not yet become routine in sports medicine clinics in the US. There is ongoing research on a number of genetic polymorphisms associated with athletic performance, but studies have involved inconsistent designs and small samples [30]. Although a number of DTC companies offer genetic testing, which they claim can help parents select the best sport for their child, our respondents do not believe such testing is ready for prime time. 

To date, no US sports medicine organization has developed a statement on the use of DTC and genetic testing for athletic performance prediction, though international consortia have begun to address the topic in recent years [3]. Sports medicine physicians in both Australia and the UK have produced consensus statements that assert that, especially given the current lack of large cohort data validating strong genetic associations [31], there are “currently no scientific grounds for the use of genetic testing for athletic performance improvement, sport selection or talent identification” [2], and that coaches and athletes should avoid the direct-to-consumer tests that are available [1,2,3]. 

Our respondents are not routinely conducting any genetic tests on patients, with the exception of SCT testing—which is the only genetic test about which the majority of physicians feel they have moderate to expert knowledge. Although the majority (67%) agreed with the NCAA policy requiring SCT screening in NCAA athletes, respondents were divided about its utility in decision-making about modifying sports participation. This is consistent with the literature in which some express concern about singling out SCT for screening when factors like obesity and tobacco more significantly increase an athlete’s risk for EHI [9], and when still other factors, like inherited cardiac arrhythmia and cardiomyopathy syndromes, are rarely screened for in the US despite the fact that they are known to increase the risk of sudden death [32].

Our respondents also express some degree of familiarity with *Apoe4*. Although research has begun to reveal the association of *Apoe4* with worse long-term outcome after a concussion, there is no evidence to support using this information in choosing treatment or in determining return-to-play recommendations in the pediatric and adult populations [16,17]. Furthermore, no medical or sports society has proposed any policy recommendations encouraging *Apoe4* testing [33]. Consistent with the current state of medical knowledge, the majority of our respondents would neither test or counsel children for *Apoe4,* nor change their post-concussion return to play practice based on *Apoe4* status. 

Physician knowledge and experiences with genetic testing for other conditions or genetic variants were limited. There was some experience with four health conditions that predisposed athletes to harm (connective tissue disorders, HCM, LTQS, and cardiac arrhythmias), and the majority thought it appropriate to perform such testing on at-risk children. In contrast, virtually all thought it inappropriate to test children for genes associated with athletic performance (*ACE II, ACTN3*) at this time, and would not recommend changes in sports participation based on the results. 

While it is reassuring that AMSSM physicians are not currently performing genetic tests for athletic aptitude or using this information to guide sports recommendations, the low level of knowledge about genetic testing across the board represents an area in need of attention. It is only a matter of time before sports medicine physicians are asked about DTC testing by parents, athletes, and/or coaches and they must know what genetic tests are available and what they can and cannot do or predict. 

The main limitation of our study was the low response rate of ~10%. While this is typical for web-based surveys [34,35], it may result in participation bias. That said, our survey sample is representative of the AMSSM membership, given a comparable gender breakdown (74% male in our respondents and in the AMSSM (AMSSM data provided by Kristin Dewitt, personal communication, 17 May 2017)). Our respondents also reported a similar breakdown of specialty training (our sample had 69% Family Medicine, 12% Pediatrics, and 8% Internal Medicine compared to 71% Family Medicine, 8% Pediatrics, and 7% Internal Medicine in AMSSM’s Annual Report) [36]. To the extent that participants are often more informed about a topic than the broader population, our respondents’ self-reporting of limited knowledge may actually overestimate the genetic knowledge of the broader sports medicine community and may also overstate its experience with genetic testing—both of which would only strengthen the need for more continuing medical education on genetics for sports medicine physicians.

## 5. Conclusions

Our study provides a baseline for understanding sports medicine physicians’ attitudes towards, experiences with, and knowledge of genetic testing. Currently, few sports medicine physicians are performing genetic tests except for SCT screening. What will happen as genetic testing becomes cheaper, more accessible, and better understood remains to be seen. Sports medicine physicians in the US need education about genetics as it relates to sports and exercise in order to be prepared to competently engage with their patients and to develop sound professional organizational policies.

## Figures and Tables

**Table 1:** Demographics, N = 216.

Characteristic/Experience	n (% *)
Gender (N = 205)	
Male	151 (74)
Female	54 (26)
Ethnicity (N = 211)	
White	182 (86)
Asian	12 (6)
Hispanic/Latino	6 (3)
Mixed	5 (2)
Black	4 (2)
Other	2 (1)
Type of Residency Training (N = 212)	
Family Medicine	149 (70)
Pediatrics	25 (12)
Internal Medicine	17 (8)
Physical Medicine and Rehabilitation	11 (5)
Emergency Medicine	8 (4)
Med/Peds	2 (1)
Additional Training After Residency (N = 208 **)	
Sports Medicine Fellowship	187 (90)
Other Fellowship	7 (3)
Masters/PhD	8 (4)
No Additional Training	15 (7)
Years in Practice (N = 207)	
Still in Training	17 (8)
0 to <10 years in practice	96 (46)
10 years or greater	94 (45)
Served as Team Physician for High School Sports Team (N = 210)	199 (95)
Served as Team Physician for a College Sports Team (N = 215)	190 (88)
Served as Team physician for a Professional Sports Team (N = 215)	86 (40)
Have you sought out DTC genetic testing for yourself? (N = 211)	
No	197 (94)
Yes, generic genetic testing	11 (5)
Yes, sports-specific genetic testing	3 (1)
Have you sought out DTC genetic testing for your children? (N = 187)	
No	187 (100)
Yes	0 (0)

DTC: direct-to-consumer.* Percentages may not add up to 100 due to rounding. ** Percentages add up to >100% because eight individuals provided more than one answer.

**Table 2:** Genetic testing requests and interactions with direct-to-consumer genetic testing.

Survey Question	n (% *)
Overall knowledge about genetic testing (N = 215)	
Minimal	122 (57)
Moderate	89 (41)
Significant	4 (2)
“Is the use of multiplex genetic testing to influence **which sport a potential athlete pursues** ready for prime time?” (N = 212)	
No	207 (98)
Yes	5 (2)
“Is the use of multiplex genetic testing to influence **an athlete’s training program** ready for prime time?” (N = 211)	
No	185 (88)
Yes	26 (12)
Number of requests for athletic-performance-related genetic testing, excluding sickle cell trait, in the past year: (N = 209)	
Zero	172 (82)
One to less than 10 times	35 (17)
Ten to less than 50 times	2 (1)
Number of requests to review results of direct-to-consumer genetic testing for sports performance: (N = 210)	
Zero	165 (79)
One to less than 10 times	41 (20)
Ten to less than 50 times	3 (1)
Fifty or greater times	1 (1)

* Percentages may not add up to 100 due to rounding.

**Table 3:** Knowledge, attitudes, and practice towards sickle cell trait testing.

Survey Question	n (% *)
Level of knowledge about sickle cell trait genetic testing (N = 213)	
Minimal	33 (16)
Moderate	102 (48)
Significant	75 (35)
Expert	3 (1)
Have you ever been asked to do genetic testing to evaluate a patient for sickle cell trait? (N = 215)	
Yes	137 (64)
Yes. Referred to genetic counselor.	8 (4)
No	70 (33)
Is it appropriate to counsel/test children for sickle cell trait? (N = 214)	
Yes, any child	82 (38)
Yes, but only with family history	94 (44)
No	30 (14)
Don’t know	8 (4)
Do you support the NCAA policy of screening student athletes for sickle cell trait? (N = 211)	
Yes	142 (67)
No	64 (30)
Not aware of the policy	5 (2)
Would you recommend changes in sports participation based on the results of sickle cell trait testing? (N = 213)	
Yes	110 (52)
No	95 (45)
Don’t know enough	8 (4)
Top 3 methods used to confirm sickle cell trait status for athletes: (N = 205)	(%)
Hemoglobin electrophoresis	(37)
Sickledex	(31)
Newborn blood spot result	(19)

NCAA: National Collegiate Athletic Association. * Percentages may not add up to 100 due torounding.

**Table 4:** Knowledge, attitudes, and practice towards *Apoe4* testing.

Survey Question	n (% *)
Level of knowledge about *Apoe4* variant testing (N = 215)	
Minimal	155 (72)
Moderate	50 (23)
Significant	10 (5)
Expert	0 (0)
Have you ever been asked to do genetic testing to evaluate a patient for *Apoe4*? (N = 215)	
No	198 (92)
Yes	10 (5)
Yes. Referred to genetic counselor.	7 (3)
Is it appropriate to counsel/test children for *Apoe4* variants? (N = 213)	
No	111 (52)
Yes, any child	9 (4)
Yes, but only with family history	41 (19)
Don’t know	52 (24)
Would you do *Apoe4* testing after a patient suffers a concussion to inform their decision about continued sports participation? (N = 208)	
No	146 (70)
Yes, but only if the patient/parent requested testing	56 (27)
Yes, I would recommend	6 (3)
Would you recommend changes in sports participation based on *Apoe4* findings? (N = 211)	
No	81 (38)
Yes	23 (11)
Don’t know Enough	107 (51)
Would *Apoe4* test results change your return-to-play recommendations following a concussion? (N = 199)	
No	163 (82)
Yes	36 (18)

* Percentages may not add up to 100 due to rounding.

**Table 5:** Knowledge, experiences, and attitudes towards genetic testing for health risks and sports performance.

Survey Question	Connective Tissue Disorders	HCM	LQTS	Cardiac Arrhythmia	*COL1A1* Variants	*ACE II* Variants	*ACTN3* Variants
Have you ever been asked to do genetic testing to evaluate a patient for conditions/traits/genetic variants that may affect sports performance or injury risk? (% Yes)	45	36	20	18	2	2	2
Do you think it is appropriate to counsel/test children interested in sports for the following conditions/traits/genetic variants? (% Yes)	73	82	77	66	18	14	13
Would you recommend changes in sports participation based on findings in any of the following? (% Yes)	NA	NA	NA	NA	4	2	2

HCM: hypertrophic cardiomyopathy; LQTS: long QT syndrome; NA: not asked.

## Abbreviations

AAP	American Academy of Pediatrics
AMSSM	American Medical Society for Sports Medicine
*Apoe4*	Apolipoprotein E4
ASH	American Society of Hematology
*ACE II*	Angiotensin Converting Enzyme 2
*ACTN3*	Alpha-Actinin 3
CAP	College of American Pathologists
*COL1A1*	Collagen, Type I, Alpha 1
DTC	Direct to Consumer
EHI	Exertional Health Illness
HCM	Hypertrophic Cardiomyopathy
LQTS	Long QT Syndrome
NATA	National Athletic Trainers’ Association
SACHDNC	Secretary’s Advisory Committee on Heritable Disorders in Newborns and Children
NCAA	National Collegiate Athletic Association
REDCAP	Research Electronic Data Capture
SCT	Sickle Cell Trait
US	United States

## References

[1] Williams A., Miah A., Harris R., Montgomery H., Wackerhage H. (2012). The BASES expert statement on genetic research and testing in sport and exercise science. Sport Exerc. Sci..

[2] Human Genetics Society of Australasia (2012). 2012PSO3: Position Statement: Genetic Testing and Sports Performance. https://www.hgsa.org.au/documents/item/19.

[3] Vlahovich N., Fricker P.A., Brown M.A., Hughes D. (2017). Ethics of genetic testing and research in sport: A position statement from the Australian Institute of Sport. Br. J. Sports Med..

[4] Jones S.R., Binder R.A., Donowho E.M. (1970). Sudden death in sickle-cell trait. N. Engl. J. Med..

[5] Drehner D., Neuhauser K.M., Neuhauser T.S., Blackwood G.V. (1999). Death among US Air Force Basic Trainees, 1956–1996. Mil. Med..

[6] Kark J.A. (2008). Sickle Cell Trait and Fatal Exertional Heat Illness: Implications for Exercise-Related Death of Young Adults. http://www.dtic.mil/dtic/tr/fulltext/u2/a500648.pdf.

[7] Kark J.A., Posey D.M., Schumacher H.R., Ruehle C.J. (2010). Sickle-cell trait as a risk factor for sudden death in physical training. N. Engl. J. Med..

[8] Marshall S.W. (2010). Heat injury in youth sport. Br. J. Sports Med..

[9] Nelson D.A., Deuster P.A., Carter R., Hill O.T., Wolcott V.L., Kurina L.M. (2016). Sickle Cell Trait, Rhabdomyolysis, and Mortality among U.S. Army Soldiers. N. Engl. J. Med..

[10] Cummings D. (2008). “Rice University, NCAA Face Lawsuit over Death of Football Player.”. http://www.findingdulcinea.com/news/sports/September-08/Rice-University--NCAA-Face-Lawsuit-Over-Death-of-Football-Player.html.

[11] National Athletic Trainers’ Association (NATA) (2012). Consensus Statement: Sickle Cell Trait and the Athlete. http://static.uafortsmithlions.com/custompages/Athletic%20Training%20Forms%202015/NATA%20Sickle%20Cell%20Statement.pdf.

[12] College of American Pathologists (CAP) (2012). Statement: Sickle Cell Trait in the Athlete. http://www.cap.org/apps/portlets/contentViewer/show.do?printFriendly=true&contentReference=statements/sickle_cell_and_the_athlete_statement.html.

[13] The American Society of Hematology (ASH) (2012). Statement on Screening for Sickle Cell Trait and Athletic Participation. http://www.hematology.org/Advocacy/Statements/2650.aspx.

[14] Secretary’s Advisory Committee on Heritable Disorders in Newborns and Children (SACHDNC) (2010). Screening U.S. College Athletes for their Sickle Cell Disease Carrier Status. https://www.hrsa.gov/sites/default/files/hrsa/advisory-committees/heritable-disorders/reports-recommendations/reports/college-athletes-sickle-cell.pdf.

[15] Graham R., Rivara F.P., Ford M.A., Committee on Sports-Related Concussions in Youth (2014). Chapter 5: Consequences of Repetitive Head Impacts and Multiple Concussions. Sports-Related Concussions in Youth: Improving the Science, Changing the Culture.

[16] Carman A.J., Ferguson R., Cantu R., Comstock R.D., Dacks P.A., DeKosky S.T., Gandy S., Gilbert J., Gilliland C., Gioia G. (2015). Expert Consensus document: Mind the Gaps—Advancing research in short-term and long-term neuropsychological outcomes of youth sports-related concussions. Nat. Rev. Neurol..

[17] Kassam I., Gagnon F., Cusimano M.D. (2016). Association of the APOE-E4 allele with outcome of traumatic brain injury in children and youth: A meta-analysis and meta-regression. J. Neurol. Neurosurg. Psychiatry.

[18] Merritt V.C., Rabinowitz A.R., Arnett P.A. (2017). The Influence of the Apolipoprotein E (APOE) Gene on Subacute Post-Concussion Neurocognitive Performance in College Athletes. Arch. Clin. Neuropsychol..

[19] Ma F., Yang Y., Li X., Zhou F., Gao C., Li M., Gao L. (2013). The Association of Sport Performance with ACE and ACTN3 Genetic Polymorphisms: A systematic review and meta-analysis. PLoS ONE.

[20] Caulfield T., Borry P., Toews M., Elger B.S., Greely H.T., McGuire A. (2015). Marginally scientific? Genetic testing of children and adolescents for lifestyle and health promotion. J. Law Biosci..

[21] Guth L.M., Roth S.M. (2013). Genetic influence on athletic performance. Curr. Opin. Pediatr..

[22] Mattson C.M., Wheeler M.T., Waggott D., Caleshu C., Ashley E.A. (2016). Sports genetics moving forward: Lessons learned from medical research. Physiol. Genom..

[23] Garrett I.A., Scott R.A. (2011). No Association between ACE Gene Variation and Endurance Athlete Status in Ethiopians. Med. Sci. Sports Exerc..

[24] Vincent B., Windelinckx A., Van Proeyen K., Masschelein E., Nielens H., Ramaekers M., Van Leemputte M., Hespel P., Thomis M. (2012). Alpha-actinin-3 deficiency does not significantly alter oxidative enzyme activity in fast human muscle fibers. Acta Physiol..

[25] Wang G., Padmanabhan S., Wolfarth B., Fuku N., Lucia A., Ahmetov I.I., Cieszczyk P., Collins M., Eynon N., Klissouras V. (2013). Chapter Four: Genomics of Elite Sporting Performance: What Little We Know and Necessary Advances. Adv. Genet..

[26] Webborn N., Williams A., McNamee M., Bouchard C., Pitsiladis Y., Ahmetov I., Ashley E., Byrne N., Camporesi S., Collins M. (2015). Direct-to-consumer genetic testing for predicting sports performance and talent identification: Consensus Statement. Br. J. Sports Med..

[27] Hess G.P., Fonseca E., Scott R., Fagerness J. (2015). Pharmacogenomic and pharmacogenetic-guided therapy as a tool in precision medicine: Current state and factors impacting acceptance by stakeholders. Genet. Res..

[28] Kolata G. (2015). The Path for Precision Medicine. The New York Times.

[29] U.S. National Library of Medicine (2017). “What Is Precision Medicine?”. https://ghr.nlm.nih.gov/primer/precisionmedicine/definition.

[30] Jacob Y., Spiteri T., Hart N.H., Anderton R.S. (2018). The Potential Role of Genetic Markers in Talent Identification and Athlete Assessment in Elite Sport. Sports.

[31] Wackerage H., Miah A., Harris R.C., Montgomery H.E., Williams A.G. (2009). Genetic research and testing in sport and exercise science: A review of the issues. J. Sports Sci..

[32] Ferrari R., Parker L.S., Grubs R.E., Krishnamurti L. (2015). Sickle Cell Trait Screening of Collegiate Athletes: Ethical Reasons for Program Reform. J. Genet. Couns..

[33] McCrory P., Meeuwisse W.M., Aubry M., Cantu B., Dvořák J., Echemendia R.J., Engebretsen L., Johnston K., Kutcher J.S., Raftery M. (2013). Consensus statement on concussion in sport: The 4th international conference on Concussion in Sport, Zurich, November 2012. Br. J. Sports Med..

[34] Constant Contact Help Center (2017). Knowledge Base: Predict Survey Response Rates. http://knowledgebase.constantcontact.com/articles/KnowledgeBase/5509-predict-survey-response-rates.

[35] Cook C., Heath F., Thompson R.L. (2000). A Meta-Analysis of Response Rates in Web- or Internet-Based Surveys. Educ. Psychol. Meas..

[36] American Medical Society for Sports Medicine (AMSSM) (2017). 2016–2017 Annual Report. https://www.amssm.org/Content/pdf%20files/AMSSM2017AnnualReportFINAL.pdf.

